# Supplementary material for: Cross-linker–mediated regulation of actin network organization controls tissue morphogenesis
Source: J Cell Biol. 2019 Jun 28;218(8):2743–61. doi: 10.1083/jcb.201811127 (PMC6683744; doi:10.1083/jcb.201811127)
Supplement: Supplemental Materials (PDF) [file JCB_201811127_sm.pdf]

Supplemental material

Krueger et al., https://doi.org/10.1083/jcb.201811127

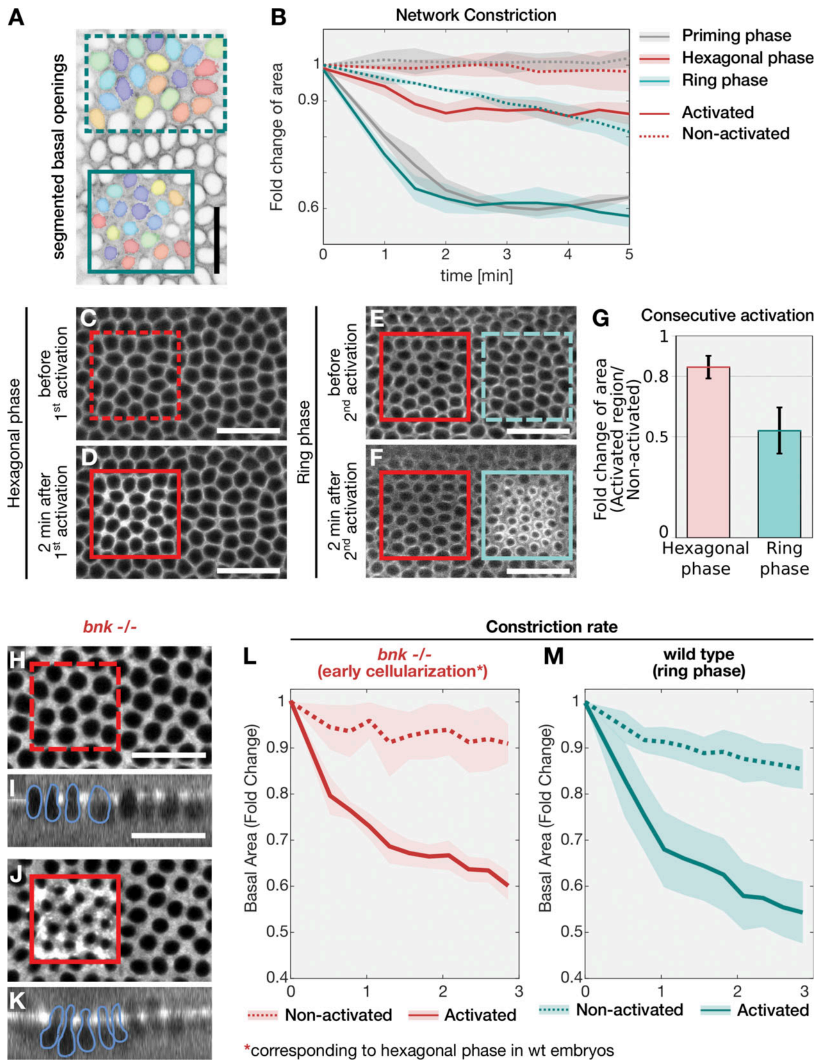

Figure S1. **Comparison of the behavior of the basal actomyosin network during different stages of cellularization upon myosin-II optogenetic stimulation. (A and B)** Cellularizing embryos coexpressing Rho-GEF2-CRY2, CIBN::GFP-pm, and the myosin-II probe Sqh::mCh were photoactivated at different stages of cellularization in a subset of cells. **(A)** Confocal section of an embryo activated during ring phase (2.5 min after photoactivation) showing segmented basal openings in the nonactivated region (dashed green box) and in the photoactivated region (solid green box). Scale bars, 10 µm. **(B)** Graph shows the quantification of the extent to which the actomyosin network constricted over time during the priming phase (gray, n = 3), hexagonal phase (red, n = 5), and ring phase (green, n = 3). The lines show the mean area of basal openings within the photoactivated region (solid, number of cells per analyzed embryo: n > 20) and in the nonactivated region (dashed, number of cells per analyzed embryo: n > 30) at the respective time points normalized to the initial time point. When myosin-II was activated during the priming or the ring phase, the network constricted significantly to ~60% of the initial value within the photoactivated region, while the nonactivated region retained the typical behavior of network stabilization, during priming phase, or constriction, during the ring phase. During the hexagonal phase, the network constricted only to a maximum of ~85% of the initial value and was stable in the nonactivated part. **(C–F)** Confocal sections of a cellularizing embryo coexpressing Rho-GEF2-CRY2, CIBN::GFP-pm, and the myosin-II probe Sqh::mCh (gray), which was photoactivated consecutively during the hexagonal (C and D) and during the ring phase (E and F). First, a subset of cells (red square/dashed line: preactivation region; red square/solid line: photoactivated region) was photoactivated using two-photon excitation (950 nm) during the hexagonal phase (C and D). Afterward, the same embryo in a different subset of cells (green square/dashed line: preactivation region; green square/solid line: photoactivated region) was photoactivated a second time during the ring phase (E and F). The red squares in E and F indicate the region of the first photoactivation. The myosin-II signal shown in C and E was recorded before photoactivation, in 2 min after the first (D) and in 2 min after the second (F) photoactivation. **(G)** Quantification of actomyosin network constriction in the photoactivated region compared with the nonactivated region upon the first photoactivation during the hexagonal phase (red) and upon the second photoactivation during the ring phase (green). Area of basal openings (n_{Hex} = 13, n_{Ring} = 17) was measured. The bar diagrams indicate the average fold change and the error bars indicate the SD. During the hexagonal phase the network constricted to an average of ~85% and during the ring phase to ~55% of the area in the nonactivated region. **(H–L)** The Rho-GEF2-CRY2/CIBN::GFP-pm optogenetic module was coexpressed with the myosin-II probe Sqh::mCh in *bnk*$^{-/-}$ embryos defective in hexagonal network patterning, and the basal actomyosin network was photoactivated at a stage corresponding to the hexagonal phase in wild-type embryos (ingression depth: 4–7 µm). **(H–K)** Confocal sections showing top views (H and J) and cross sections (I and K) of the basal actomyosin network (Sqh::mCh) before (H and I) and 2 min after (J and K) photoactivation. Scale bars, 20 µm. The shape of nuclei in the activation region was highlighted in blue in I and K. **(L and M)** Graphs showing network constriction in *bnk*$^{-/-}$embryos ($n_{bnk-/-}$=5) photoactivated at a stage corresponding to the hexagonal phase in wild-type embryos and in control embryos ($n_{Control}$ = 7) during the ring phase in the nonactivated region (dashed line) and photoactivated region (solid line). Lines indicate the mean area of basal openings over time; semitransparent regions indicate the SD. Photoactivation of *bnk*$^{-/-}$ embryos at a stage corresponding to the hexagonal phase in wild-type embryos causes a similar contractile response as during the ring phase in control embryos.

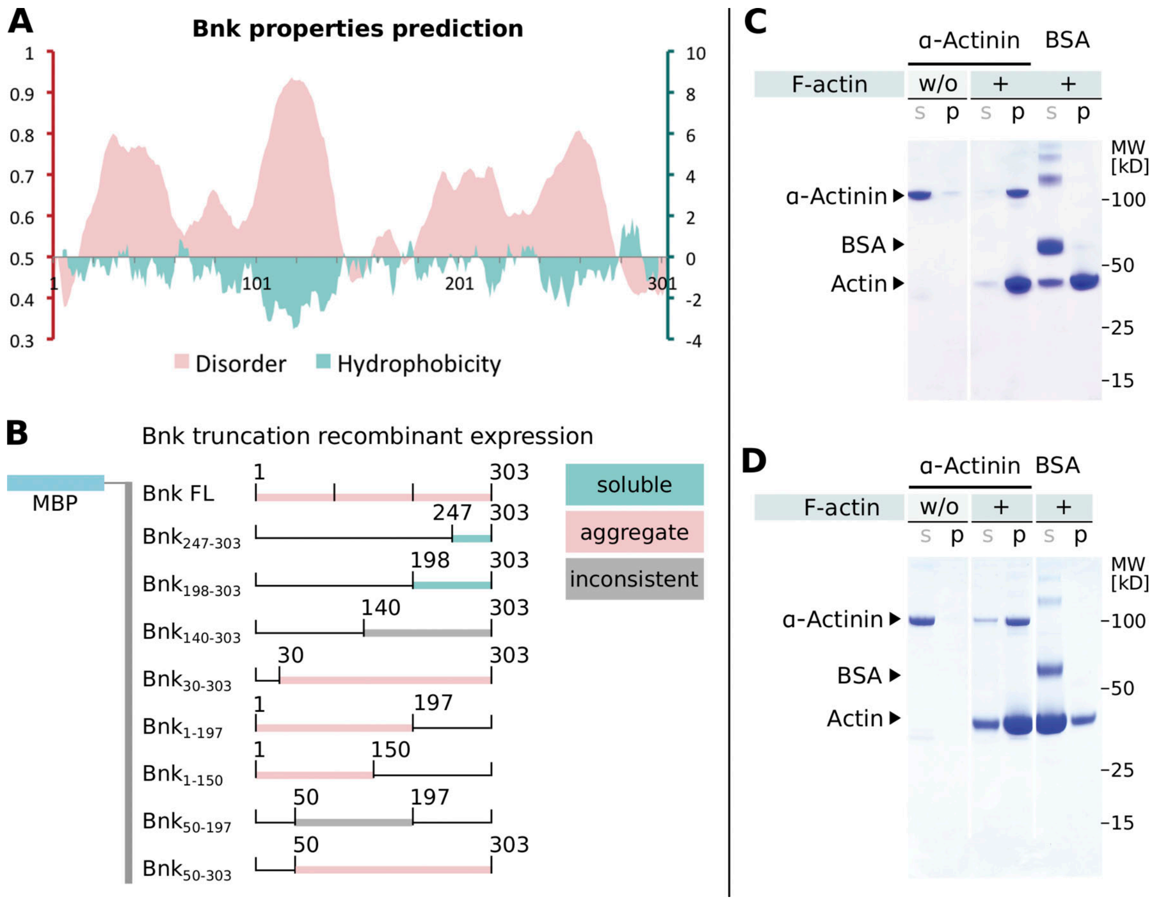

Figure S2. **Bnk is an intrinsically disordered and hydrophobic protein. (A)** Graph showing a per-residue disorder prediction (red, left y axis, RONN v3.2) and a Kyte–Doolittle hydrophobicity plot (green, right y axis, ExPASy ProtScale) of the 303–amino acid–spanning Bnk. Bnk is highly unstructured (score >0.5 indicates disorder) and hydrophobic (high Kyte-Doolittle score indicates high hydrophobicity). **(B)** Overview of the *bnk* fragments expressed in *E. coli* and purified as MBP fusion proteins. Red color indicates that the fusion protein was present in protein aggregates (insoluble); green color indicates that the fusion protein was present in a nonaggregated (soluble) state as assessed by size exclusion chromatography. Gray color indicates that the obtained results were inconsistent, suggesting protein instability. FL, full length. **(C)** Coomassie gel showing the results of an actin-binding assay performed using α-actinin and BSA. In the absence of F-actin, α-actinin was present in the supernatant fraction (s), whereas in the presence of F-actin, the majority of α-actinin but not BSA moved to the pellet fraction (p). **(D)** Coomassie gel showing the results of an actin-bundling assay performed on the control proteins α-actinin and BSA. α-Actinin moved from the supernatant fraction in absence of F-actin into the pellet fraction in the presence of F-actin. BSA remained in the supernatant faction in the presence of F-actin. The ratio of F-actin in the pellet to that in the supernatant fraction increased in the presence of α-actinin but not in the presence of BSA.

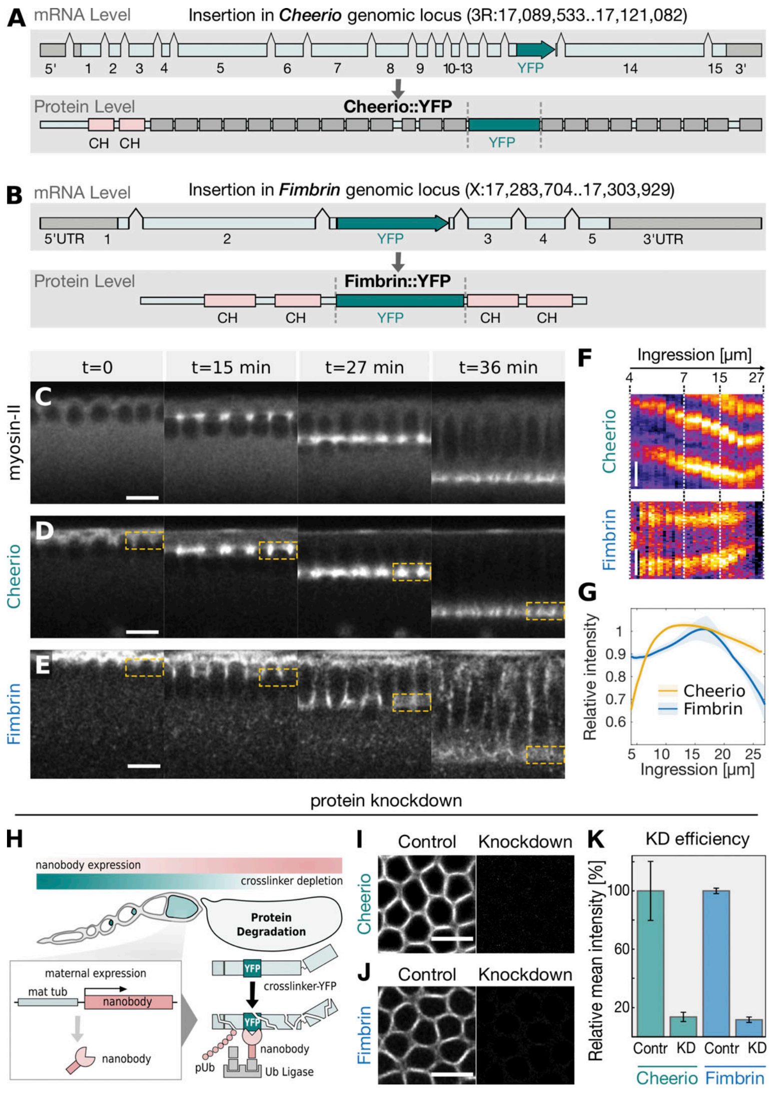

Figure S3. **Map showing the position of the YFP-tag in *cheerio* and *fimbrin* and the localization of the proteins to the leading edge of the cellularization furrow. (A and B)** Schematic representation of the YFP gene trap, comprising the YFP/Venus gene flanked by a splice donor and splice acceptor site, inserted into the genomic locus of *cheerio* (A) and *fimbrin* (B) at the mRNA level (upper panel) and protein level (lower panel). mRNA architecture of the tagged genes. Exons are numbered and shown in light green, UTRs are shown in gray, the YFP gene is shown in dark green, and introns are shown as lines connecting the exons (upper panels in A and B). Protein architecture coded by the tagged genes. The actin-binding Calponin homology (CH) domains are shown in light red, and the fluorescent protein YFP is shown in dark green. Ig-like FLN repeats are shown in dark gray (lower panels in A and B). Dashed lines indicate the splice sites flanking the YFP-insertion. The YFP-tagged fly lines cher[CPTI001399] and fim[CPTI100066] were obtained from the CPTI collection. The insertion sites for fim[CPTI100066] were characterized here for the first time. **(C–E)** Confocal (C and D) and two-photon (E) microscopy images of cellularizing embryos expressing the myosin-II probe Sqh::mCh (C), Cheerio::YFP (D), or Fimbrin::YFP (E). Still frames showing the sagittal cross sections (from left to right) at the onset of cellularization (t = 0), and after 15 min, 27 min, and 36 min. Similar to myosin-II (C), Cheerio localized predominantly to the cellularization front (D) throughout cellularization. Fimbrin was enriched at the cell base throughout cellularization but also localized to other sites of the cell (E). Yellow boxes in D and E correspond to the region which were used to generate the kymographs presented in F. Scale bars, 20 µm. **(F)** Kymograph showing Cheerio::YFP (upper panel) or Fimbrin::YFP (lower panel) intensity at the cell base (corresponding to yellow boxes in D and E) over the course of cellularization (40 min). White dashed lines indicate the respective ingression depth of the cellularization furrow. Image sections were pseudo-colored with the fire lookup table (LUT, ImageJ software). Scale bar, 2 µm. **(G)** Graph showing the relative abundance of Cheerio and Fimbrin at the cell base as a function of ingression depth of the cellularization furrow. Embryos expressing endogenously YFP-tagged Cheerio (n = 4) or Fimbrin (n = 4) were imaged throughout cellularization; the fluorescence intensity at the cell base was measured and plotted as a function of ingression depth. Lines indicate mean intensity and semitransparent regions indicate the SD. **(H)** Cartoon schematizing the nanobody-mediated protein KD approach employed to induce Cheerio and Fimbrin loss-of-function phenotypes during early embryonic development. The anti GFP-nanobody was expressed under the control of the maternal tubulin promoter (mat tub) restricting its expression to the developing oocyte. The nanobody recognizes YFP, and upon binding to the endogenously tagged cross-linker, the coupled F-box protein induces recruitment of the endogenous ubiquitin ligase (Ub ligase) to prime the targeted cross-linker for proteasomal degradation by poly-ubiquitinylation (pUb). This approach resulted in efficient degradation of the YFP-tagged cross-linker in the early embryo while maintaining residual protein levels to support oogenesis. **(I and J)** Representative confocal images showing Cheerio::YFP (I) and Fimbrin::YFP (J) in control or upon nanobody expression (KD). **(K)** Bar diagrams showing protein levels of Cheerio (green) and Fimbrin (blue) measured based on the YFP-fluorescent signal in control (Contr) and upon protein KD and normalized to the control condition. Fluorescence was normalized to the mean signal of control embryos ($n_{\text{Control Cheerio}}$ = 34, $n_{\text{Cheerio KD}}$ = 29, $n_{\text{Control Fimbrin}}$ = 12, $n_{\text{Fimbrin KD}}$ = 24), and error bars show the SD.

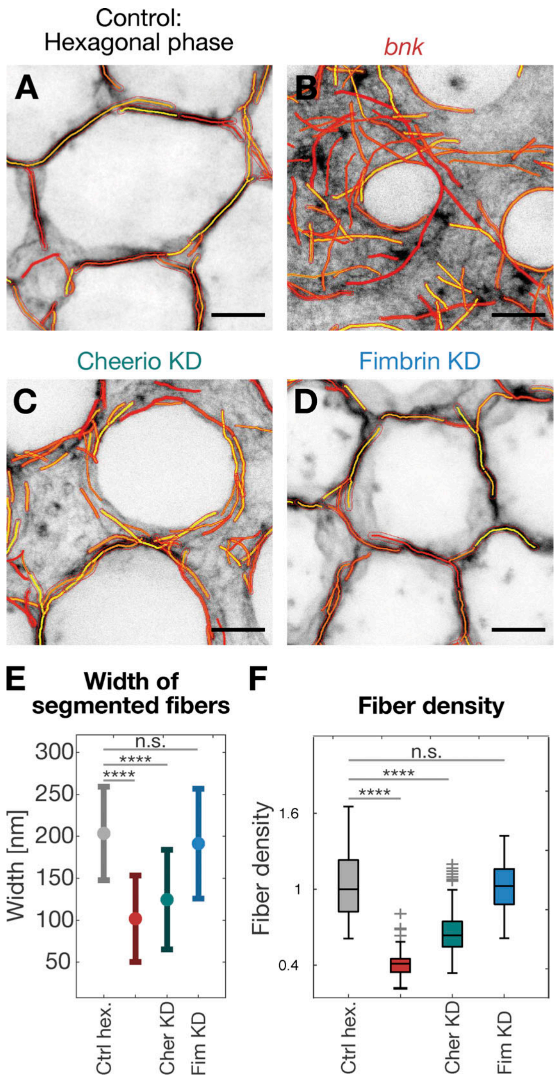

Figure S4. **Quantification of actin fibers width and density from STED images. (A–D)** *Drosophila* embryos stained with phalloidin–Atto647N and imaged by 2D-STED microscopy (wild-type hexagonal phase [A], $bnk^{-/-}$ [B], Cheerio KD [C], and Fimbrin KD [D]; corresponding to data presented in Fig. 5, B, H, K, and Q, respectively) were subjected to fiber segmentation using SFEX (Zhang et al., 2017). Inverted color STED images were overlaid with colored segmented fibers; red outlines indicate fiber width. Scale bars, 2 µm. **(E and F)** Graphs showing the width (E) and density (F) of segmented fibers in control embryos during hexagonal phase (Ctrl hex.; n_{Control} = 89), $bnk^{-/-}$ ($n_{bnk\ -/-}$ = 241), Cheerio KD (Cher KD; n_{CherKD} = 202), and Fimbrin (Fim KD, n_{FimKD} = 129) KD embryos. In each box plot, the central mark, the bottom, and the top edge indicate the median and the 25th and 75th percentiles, respectively. Whiskers extend to the most extreme data point. **(F)** Fiber density was calculated from the phalloidin intensity of segmented fibers divided by the intensity of a single fiber. The obtained values were normalized to the median of the control embryo during hexagonal phase. For all panels, when indicated, significances were estimated using nonpaired Student's *t* test with ****, $P < 0.0001$; n.s., not significant.

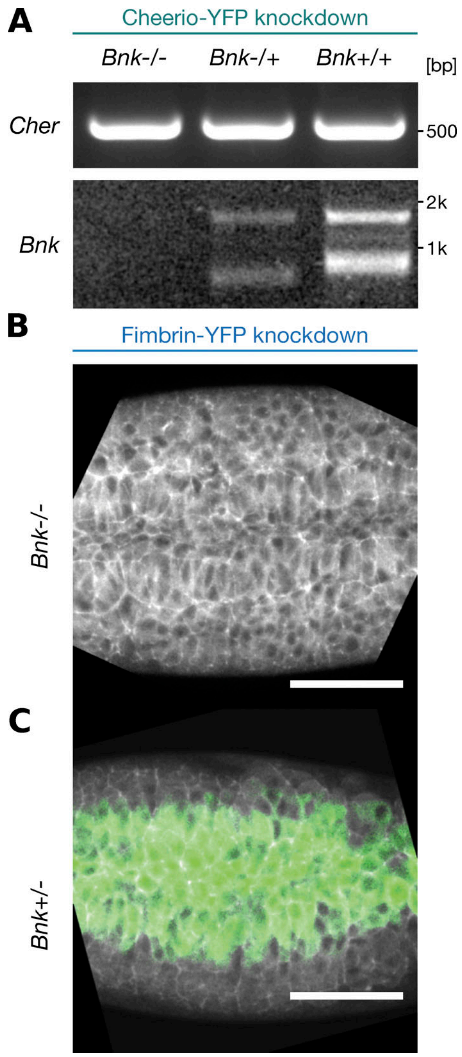

Figure S5. **Verification of the *bnk* mutant genotype in Cheerio and Fimbrin KD embryo by PCR or GFP reporter expression. (A)** Agarose gel electrophoresis image of a PCR genotyping using primer pairs specific for *cheerio* or *bnk*. After time-lapse imaging, *bnk*$^{-/-}$ and Cheerio KD double mutant embryos were genotyped by PCR. *bnk*$^{-/-}$ embryos lacked a specific expected ∼900-bp band found in heterozygous (*bnk*$^{-/+}$) or wild-type (*bnk*$^{+/+}$) embryos, while the *cheerio*-specific PCR did not show any variation in the expected ∼550-bp PCR product. **(B and C)** Confocal images of *Drosophila* embryos expressing the nanobody KD module targeting Fimbrin, the myosin-II probe Sqh::mCh (gray), and cytoplasmic GFP (green; in *bnk*$^{+/-}$ or *bnk*$^{+/+}$ embryos) at gastrulation. The parental generation of *bnk*$^{-/-}$ Fimbrin KD double mutant embryos carried the *bnk* deficiency over a TM3, Ser, Sb balancer chromosome that also contained the GAL4 transcription factor expressed under the twist promoter (*ptwist*>GAL4) and a UAS::GFP transgene. *bnk*$^{-/-}$ Fimbrin KD double mutant embryos were identified by the lack of GFP expression (B), as heterozygous (*bnk*$^{-/+}$) or homozygous positive (*bnk*$^{+/+}$) embryos expressed GFP during gastrulation in the ventral cells (C). Scale bars, 50 µm.

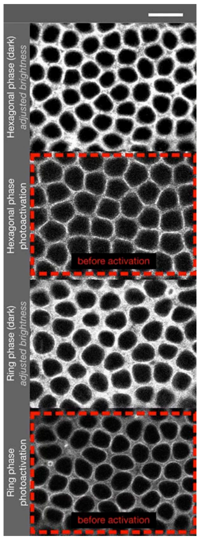

Video 1. **Actomyosin network configurations display differential contractile responses upon optogenetic myosin-II stimulation and subseqent laser ablation.** Confocal video of *Drosophila* embryos coexpressing Rho-GEF2-CRY2, CIBN::GFP-pm and the myosin-II probe Sqh::mCh. Embryos were imaged using a 561-nm laser to visualize the actomyosin network (grayscale) during the hexagonal phase (from top: first and second panel) or during the ring phase (from top: third and fourth panel). While the first and third (from top) embryos were only imaged using 561-nm light, the second and fourth (from top) embryos were photoactivated at the cell base (red box; dashed lines indicate region of photoactivation before activation) using 950-nm two-photon light before the actomyosin network was ablated. After laser ablation (indicated by red line), displacement of the basal surface was tracked manually; colored dots and lines show corresponding tracking paths. Note that the contrast of the nonactivated embryos (from top: first and third) was adjusted to match similar intensity levels after laser ablation. Frames were collected every second; display rate: 33 frames/second. Scale bar, 10 µm.

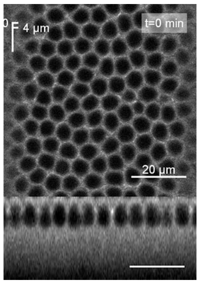

Video 2. **The basal actomyosin network during cellularization undergoes a transition from a hexagonal to a round network.** Confocal video of a control embryo expressing the myosin-II probe Sqh::mCherry (grayscale) showing the basal actomyosin network from a top view (upper panel) or in a sagittal section (lower panel). The ingression depth of the cellularization furrow is indicated in the top left. The network assembled into a hexagonal network and after ingressing for 7 µm rounded up into individualized contractile rings. Frames were collected every 1.5 min; display rate: 5 frames/second. Scale bars, 20 µm.

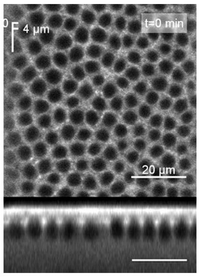

Video 3. **The basal actomyosin network is disorganized in a *bnk*$^{-/-}$ embryo and undergoes premature constrictions.** Confocal videos of *bnk*$^{-/-}$ embryo expressing the myosin-II probe Sqh::mCherry (grayscale) showing the basal actomyosin network from a top view (upper panel) or in a sagittal section (lower panel). The ingression depth of the cellularization furrow is indicated in the top left. The actomyosin network did not acquire a hexagonal configuration constricted prematurely deforming trapped nuclei into bottleneck-like shapes. Frames were collected every 1.2 min; display rate: 5 frames/second. Scale bars, 20 µm.

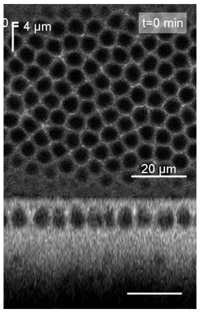

Video 4. **The hexagonal phase of cellularization is prolonged in Fimbrin KD embryos.** Confocal video of Fimbrin KD embryo expressing the myosin-II probe Sqh::mCherry (grayscale) showing the basal actomyosin network from a top view (upper panel) or in a sagittal section (lower panel). The ingression depth of the cellularization furrow is indicated in the top left. The hexagonal phase of cellularization was prolonged and the basal actomyosin network showed angular characteristics even at an ingression depth of 15 µm. Frames were collected every 0.9 min; display rate: 5 frames/second. Scale bars, 20 µm.

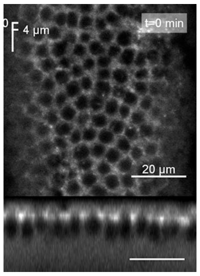

Video 5. **The basal actomyosin network does not assemble into a hexagonal pattern upon KD of Cheerio.** Confocal video of a Cheerio KD embryo expressing the myosin-II probe Sqh::mCherry (grayscale) in showing the basal actomyosin network from a top view (upper panel) or in a sagittal section (lower panel). The ingression depth of the cellularization furrow is indicated in the top left. The basal actomyosin network lacked the hexagonal phase and displayed a meshwork-like organization. Frames were collected every 1.2 min; display rate: 5 frames/second. Scale bars, 20 µm.

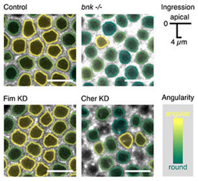

Video 6. **Comparison of actomyosin network angularity in Fimbrin KD versus Cheerio KD and *bnk*$^{-/-}$ embryos.** Confocal video of embryos expressing the myosin-II probe Sqh::mCherry (grayscale) showing the basal actomyosin network from a top view in a control (top left), a *bnk*$^{-/-}$ (top right), a Fimbrin KD (bottom left) and a Cheerio KD (bottom right) embryo. Basal openings were segmented and colored according to the respective angularity value (high angularity: yellow; low angularity/round: green). The basal networks were compared based on the ingression depth of the cellularization furrow, which is indicated in the top right of each panel. While bnk$^{-/-}$ and Cheerio KD embryos lacked the hexagonal phase, Fimbrin KD embryos showed a prolonged and more pronounced hexagonal pattern. Display rate: 1 frame/second. Scale bars, 20 µm.

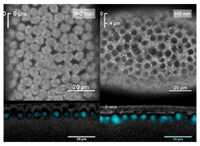

Video 7. **The ingression of the cellularization furrow is arrested in *bnk*$^{-/-}$ Cheerio KD double mutant embryos.** Confocal video of a control (left panels) and a *bnk*$^{-/-}$ Cheerio KD double mutant embryo (right panels) expressing the myosin-II probe Sqh::mCherry (grayscale) showing the top view (upper panels) or in a sagittal section (lower panels). The position of the nuclei (cyan) was inferred from the lack of myosin-II signal. In the control embryo, cells reached a length of ∼30 µm after ∼45 min of cellularization. In the *bnk*$^{-/-}$ Cheerio KD double mutant embryo, the disorganization and premature contraction of the actomyosin network caused an arrest of cellularization apically of the nuclei. Frames were collected every 1.2 min; display rate: 4 frames/second. Scale bars, 20 µm.

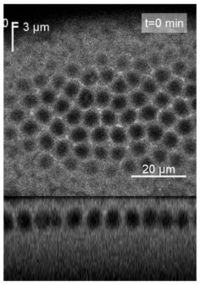

Video 8. **KD of Fimbrin in a *bnk*$^{-/-}$ embryo rescues actomyosin network organization and premature constriction.** Confocal video of a *bnk*$^{-/-}$ Fimbrin KD double mutant embryo expressing the myosin-II probe Sqh::mCherry (grayscale) showing the basal actomyosin network from the top view (upper panel) or in a sagittal section (lower panel). The ingression depth of the cellularization furrow is indicated in the top left. The disorganization and premature contraction of the *bnk* mutant phenotype was rescued by the concomitant reduction of Fimbrin. Frames were collected every 3.3 min; display rate: 2 frames/second. Scale bars, 20 µm.

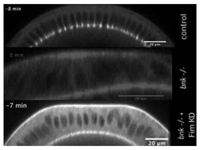

Video 9. **KD of Fimbrin in a *bnk*$^{-/-}$ embryo rescues ventral furrow formation.** Confocal videos (cross section) of a control embryo (upper panel), a *bnk*$^{-/-}$ mutant embryo (middle panel), and a *bnk*$^{-/-}$ and Fimbrin KD double mutant embryo (lower panel) expressing the myosin-II probe Sqh::mCherry (grayscale). In a control embryo, the process of ventral furrow formation was completed in ∼15 min, while the *bnk*$^{-/-}$ mutant embryo failed to complete ventral furrow formation. KD of Fimbrin in a *bnk*$^{-/-}$ embryo rescued ventral furrow formation. For all videos, time 0 corresponds to the initial accumulation of myosin-II at the apical surface of ventral cells. Frames were collected at an interval of 1.4 min (upper panel), 0.7 min (middle panel), and 2.3 min (top panel) with a display rate of 4 frames/second (upper panel), 8 frames/second (middle panel), and 2.5 frames/second (top panel). Scale bars, 20 µm.

Reference

Zhang, Z., S. Xia, and P. Kanchanawong. 2017. An integrated enhancement and reconstruction strategy for the quantitative extraction of actin stress fibers from fluorescence micrographs. *BMC Bioinformatics*. 18:268. https://doi.org/10.1186/s12859-017-1684-y
